# Determinants of Fish Intake and Complying with Fish Consumption Recommendations—A Nationwide Cross-Sectional Study among Secondary School Students in Poland

**DOI:** 10.3390/nu16060853

**Published:** 2024-03-15

**Authors:** Zofia Utri-Khodadady, Dominika Skolmowska, Dominika Głąbska

**Affiliations:** Department of Dietetics, Institute of Human Nutrition Sciences, Warsaw University of Life Sciences (WULS-SGGW), 159C Nowoursynowska Street, 02-776 Warsaw, Poland; zofia_utri@sggw.edu.pl (Z.U.-K.); dominika_skolmowska@sggw.edu.pl (D.S.)

**Keywords:** fish consumption, food intake determinants, eating behaviors, recommendations, nutritional knowledge, teenagers, adolescents, parents/guardians

## Abstract

Fish intake in youth is commonly inadequate with several potential determinants. This cross-sectional study aimed to assess the influence of potential fish intake determinants in a nationwide sample of Polish youth. Associations between the participants’ fish intake and their gender, age, body mass index, place of residence (region and size of locality), school type, nutritional knowledge about fish, and their parents’/legal guardians’ fish intake were assessed. A total sample of 1317 adolescents (870 female, 447 male) aged 14–22 from 32 secondary schools from all regions of Poland participated in the study. Median fish intake among the youth was 34.9 g/week. The recommendation to consume at least 300 g of fish/week was followed by 6% of participants. Fish intake was determined by gender and the type of school, with males and comprehensive high school students consuming more fish, but it was not determined by the region or size of the locality of residence and age group, nor did the body mass index determine fish intake. Participants’ fish intake was positively associated with their nutritional knowledge about fish, as well as with their parents’/legal guardians’ fish intake. Most youths do not follow the recommendation to consume at least 300 g of fish weekly; hence, nutritional education on the recommendations and the benefits of fish consumption is necessary.

## 1. Introduction

Adolescence is a vital period in life as this is when numerous systems, such as the neurological system, the reproductive system, and the endocrine system are developed [1]. It is also a time of transition when habits are created, which can persist in adult life [2]. Moreover, the peak bone mass is initiated and achieved during puberty and early adulthood [3]. Nutrition, and consequently, nutritional status in childhood and early adolescence, influences the timing of puberty and has consequences on the proper development of the above-mentioned systems [4]. Therefore, not only should nutrition during adolescence provide all the necessary nutrients to meet the demands of physical and cognitive growth but it also ought to encourage and promote healthy dietary habits and lifestyle [5].

Fish contain high-quality protein as well as other essential nutrients of metabolic and hormonal importance, including *n*-3 polyunsaturated fatty acids, minerals (iodine, selenium), and vitamin D [6]. Fish consumption is associated with several significant health benefits. In an umbrella review of meta-analyses, it was found that high fish intake was associated with various beneficial health outcomes, including decreased all-cause death rate, decreased risk of different types of cancers (including colorectal, lung, hepatocellular, and oral cancer), and reduced risk of some cardiovascular diseases, such as the acute coronary syndrome, heart failure, and stroke [7]. Another one showed that fish consumption was also associated with a reduced risk of depression [8]. Last but not least, a recent German cohort study found that consuming fish is positively associated with school performance in children [9].

Due to its multiple health benefits, fish is usually recommended as part of a healthy balanced diet in most dietary guidelines [10,11,12,13,14], including those for children and adolescents [15,16,17]. Interestingly, some nutritional recommendations differ from country to country, and according to the World Health Organization, they often reflect the national dietary habits [18]. This is also observed in the case of fish intake recommendations. The Austrian nutritional recommendations for children indicate one to two portions of fish, ideally oily [10], to be consumed in a week [15], with a portion size of 150 g [10]. According to the British National Health Service (NHS), a proper diet should include at least two portions of fish a week (corresponding to around 280 g a week), including one portion (around 140 g) of oily fish, such as herring, salmon, mackerel, or trout [12]. The American Food and Drug Administration (FDA) advises children who are 11 years old and older, as well as adults, to consume two to three servings of different fish a week, while a typical serving corresponds to approximately 4 ounces (113 g) [16]. As for the Polish recommendations for children and adolescents, fish should be consumed at least twice a week [17], while the recommended portion is 150 g [19]. Based on the above recommendations, the recommended fish intake corresponds to around 150–300 g weekly [10,12,16,19].

Global data indicate that fish intake varies greatly between countries [20], which is associated with a very diverse consumption of *n*-3 fatty acids of seafood origin, including fish, with only 18.9% of the global population achieving the recommended intake of at least 250 mg per day [21]. The Polish Food and Nutrition Institute [22] emphasized that Poland, when compared with other European Union countries, is among countries characterized by low fish consumption, which is confirmed by the European Market Observatory for Fisheries and Aquaculture (EUMOFA) [23]. 

It must be noted that some methods of obtaining worldwide dietary data for monitoring nutrition, such as household budget surveys or balance sheets, do not accurately reflect the food intake of individuals [24]. In Poland, the available data concerning fish intake as well as the analyses and recommendations are usually based on balance sheets or household budget surveys. Some studies, such as the European Prospective Investigation into Cancer and Nutrition (EPIC), which collected data from 1992 to 2000 [25], did assess fish intake in some European countries with the use of the 24 h dietary recall, which might be more accurate than the household budget surveys or balance sheets, but the analysis did not include Poland. Up to now, there are no comprehensive data concerning fish intake in the Polish population. Therefore, it is crucial to conduct well-designed nationwide studies to accurately assess food intake in vulnerable populations, such as adolescents. 

Food choices made by adolescents are especially important, as the food intake in this period may be a determinant both of their current health status and their health status in adulthood [26]. There are various factors influencing food choices and food intake—both internal and external. Internal determinants include physiological needs, self-image, individual health, and preferences, while external factors are associated with family habits, social and cultural values, media, and friendship [27]. Importantly, consumers’ attitudes toward nutrition and food may be an important factor affecting dietary behaviors and food intake [28]. Among all the multiple food choice determinants observed in the group of adolescents, the importance of parental attitudes and parental dietary knowledge is often highlighted [29]. In the systematic review of Liu et al. [29], it was shown that these two factors may promote adolescents’ knowledge, attitude, and practice of healthy eating. Although some studies describe the determinants of fish consumption in a group of children [30,31], studies focused on youth are scarce. 

Therefore, taking all the above into account, the present cross-sectional study aimed at the assessment of fish intake and its determinants in a nationwide sample of Polish youth aged 14–22 years. 

## 2. Materials and Methods

### 2.1. Ethical Statement 

The study was carried out at the Department of Dietetics, Warsaw University of Life Sciences (WULS-SGGW) and was conducted according to the guidelines laid down in the Declaration of Helsinki. All participants, as well as their parents/legal guardians provided informed consent to participate. All procedures involving human subjects received the approval of the Ethics Committee of the Central Clinical Hospital of the Ministry of Interior and Administration in Warsaw (2/2021). The data for the study were collected from May to July 2021.

### 2.2. Studied Group

In order to gather a nationwide sample, the cross-sectional study was conducted among Polish secondary school students attending one of the five types of schools: comprehensive high schools, specialized high schools, vocational schools, technical schools, and visual arts high schools, for which the net enrollment rate in Poland is 90.83% [32]. The list of schools for the sampling was derived from the online National Register of Schools and Educational Establishments of the Polish Ministry of Education and Science [33]. The stratified random sampling method was implemented to invite students to take part in the study, and it consisted of two stages: (1) random sampling of 30% of counties from each of the sixteen Polish voivodeships (basic administrative units of Poland), and (2) inviting students from all the secondary schools from the sampled counties. In total, 115 counties were sampled and students from 1357 secondary schools were invited to take part in the study. Students were to provide data about themselves as well as about their parents/legal guardians. 

An invitation email with the aim and the scope of the study was sent to the headmasters and/or the school secretaries of the sampled schools. In the case of a lack of response, the email was resent. In the end, students from 32 secondary schools participated in the study. 

Headmasters and/or the school secretaries of the participating schools were sent an electronic link to the study questionnaire prepared in Google Forms, which was then passed along to the students. Along with the questionnaire guidelines on how to carry the study out (e.g., that the parents/legal guardians, as well as teachers, should not help the students fill in the questionnaire, and that it is possible to fill it in on a mobile phone, but it is more comfortable to fill it in with the use of a computer) were also provided. Taking part in the study was voluntary, and the dedicated questionnaire was anonymous and did not collect any data that would allow the identification of the respondents. However, the questionnaire allowed the verification of the inclusion and exclusion criteria. 

The inclusion criteria were as follows:

- Age: 14–22 years;
- Attending one of the five given types of secondary school in Poland: comprehensive high school, specialized high school, vocational, technical, or visual arts high school;
- Attending a secondary school sampled within the study;
- Informed consent to participate (verified by the headmaster);
- Informed consent of parent/legal guardian for participation (verified by the headmaster).

The exclusion criteria were as follows:

- Any missing data within the questionnaire completed;
- Any unreliable answers within the questionnaire completed.

**Figure 1:**
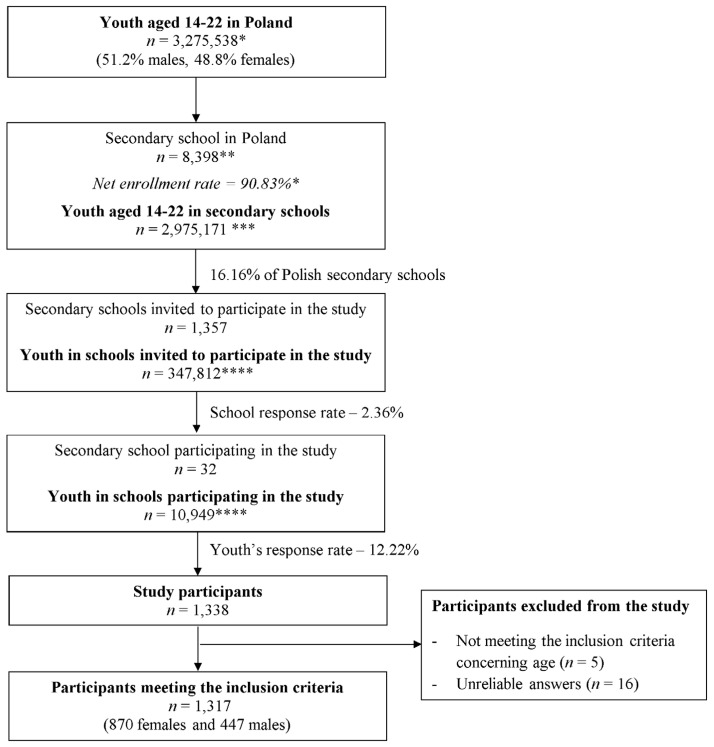
Flow diagram of the sampling procedure. * based on Statistics Poland data for the year 2021 [32], ** based on the National Register of Schools and Educational Establishments of the Polish Ministry of Education and Science for the year 2021 [33], *** calculated based on Statistics Poland data for the year 2021 [32], **** calculated based on the National Register of Schools and Educational Establishments for the year 2021 of the Ministry of Education and Science [33].

The total sample of students who completed the questionnaire comprised 1317 students from all seven macroregions in Poland (NUTS 1 units in the statistical division of Poland from the year 2021 [34]). In total, the study was conducted in 32 secondary schools, which does not differ from a typical response rate for studies conducted in Poland based on the national school sampling procedure and is comparable with the response rate observed by Głąbska et al. [35]. Such response rate, which is lower than expected, is by other authors indicated to be caused by the fact that some headmasters or teachers do not want to have their classes interrupted in any way, which results in rejecting to participate in a study by the school [36]. The detailed sampling procedure is presented in Figure 1. 

### 2.3. Applied Questionnaire and Data Analysis

The applied questionnaire used for the study consisted of four parts. The first part included basic close-ended questions about the participant’s gender, region of residence, size of the locality of residence, and type of school attended; and open-ended questions about the participant’s age, height, and weight.

The second part of the questionnaire comprised questions needed to estimate the fish and fish products intake of the participant. Four questions were included in this part: (1) ‘How often do you consume fish?’—a close-ended question with examples of various fish dishes listed (such as baked, fried, cooked, fish fingers, and fish cutlets) with five possible answers (never, less than once a month, one to three times a month, one to two times a week, three or more times a week); (2) ‘How big (in grams) is usually the fish portion you consume?’—an open-ended question where only digits and numbers were accepted; (3) ‘How often do you consume fish products?’—a close-ended question with examples of various fish products listed (such as canned fish, smoked fish, fish in cream or oil sauce, and fish spreads) with five possible answers (never, less than once a month, one to three times a month, one to two times a week, three or more times a week); and (4) How big (in grams) is usually the fish product portion you consume?’—an open-ended question where only digits and numbers were accepted. Questions 1 and 3 were based on questions from a short food frequency questionnaire to assess intake of seafood and *n*-3 supplements, which was validated with biomarkers in adults by Dahl et al. [37], while questions 2 and 4 were accompanied by exemplary pictures of fish and fish products from the Polish Food Composition Tables [38] to facilitate the exact estimation of the typically consumed portion. In question 2, nine pictures showing the portion sizes of exemplary fish dishes were presented: one fish finger (25 g), two fish fingers (50 g), three fish fingers (75 g), a small portion of a baked whole fish (90 g), a medium-size portion of a baked whole fish (180 g), a big portion of a baked whole fish (270 g), a small portion of a fried fish fillet (85 g), a medium-size portion of a fried fish fillet (170 g), and a big portion of a fried fish fillet (225 g). In question 4, nine pictures showing the portion sizes of exemplary fish products were again presented: a herring rollmop (50 g), a medium-sized portion of smoked salmon (50 g), a medium-sized portion of canned tuna (50 g), a small smoked sardine-like fish (20 g), a medium-size sardine-like fish (30 g), and a big sardine-like fish (50 g), half of a smoked mackerel (100 g), three-quarters of a smoked mackerel (150 g), and a whole smoked mackerel (200 g).

To estimate fish consumption, the qualitative answers to questions (1) ‘How often do you consume fish?’ and (3) ‘How often do you consume fish products?’ were transformed into quantitative—to represent the estimated number of portions of fish consumed in a week. The answer ‘never’ corresponded to 0 portions of fish in a week, the answer ‘less than once a month’ corresponded to 0.116 (an average of half a portion of fish in a month divided by 4.3—the average number of weeks in a month), the answer ‘one to three times a month’ to 0.465 (an average of two portions of fish in a month divided by 4.3—the average number of weeks in a month), and the answer ‘one to two times a week’ to 1.5 and ‘three or more times a week’ to 3 portions of fish in a week. To estimate the weekly fish intake in grams, the calculated estimated number of portions consumed in a week based on question 1 was multiplied by the typical fish portion the respondents indicated in question 2. Similarly, to estimate the weekly fish products intake in grams, the calculated estimated number of portions consumed in a week based on question 3 was multiplied by the typical fish products portion the respondents indicated in question 4. To estimate total weekly fish consumption, the two above-described sums were totaled up. For the analyses, the weekly total fish consumption was used—the sum of the weekly consumed fish and fish products.

In the third part of the questionnaire, students were asked about their parents’/legal guardians’ fish intake. First, questions on their parent’s/legal guardian’s gender (close-ended question) and age (open-ended question) were posed, as well as whether it is a parent, a different relative, or a non-related legal guardian. They were followed by questions concerning the parent’s/legal guardian’s estimated fish and fish products intake. The whole process of this estimation was analogous to the one undertaken for the child (the second part of the questionnaire). Just like in the case of the children for the analyses, the total weekly fish intake of one person was used—the sum of fish and fish products.

Pupils were asked whether they lived with only one or two parents/legal guardians; in the case of living with two, they were asked to estimate the fish and fish products intake for both parents/legal guardians. For data analyses, if a student indicated one female and one male parent/legal guardian, both were taken into consideration; if a student indicated both female or both male parents/legal guardians, only data for the first one indicated were taken into consideration; while if a student indicated both female or both male parents/legal guardians and the first one was not related to the student, while the second was, data for the second one indicated were taken into consideration. 

The fourth part of the questionnaire consisted of a short close-ended twenty-statement-long fish knowledge test concerning fish intake benefits and safety risks based on questions and answers from a study by Burger and Gochfeld [39]. The test included 12 true and 8 false statements and covered the following issues: the content of nutrients in fish (statements 1–5, 9, 11, 19), their influence on human health (statements 6–8, 10), the health risks associated with consuming fish (statements 12, 13, 16, 18, 20), and the nutritional recommendations concerning fish (statements 14, 15, 17). To reduce the question order bias, the statements were intentionally put in a disorganized order, with not all statements concerning one issue consecutively. Also, to reduce the confirmation bias, the statements were intentionally formulated in a way to include a similar number of correct and false statements as presented in the recently published study [40]. Students were asked to evaluate each statement whether in their opinion it was true or false; they could choose the ‘I don’t know’ answer as well. For each correct answer, they received 1 point, and for each false or ‘I don’t know’ answer, they received 0 points, thus the possible range of points was 0 to 20 points. The fish knowledge test used in the study is presented in Table 1. 

Prior to the main research a pilot study among 28 students from two different schools, which did not take part in the main study, was conducted in order to ensure that all questions were comprehensible and that no technical problems would occur. It confirmed that the questions in the study were understandable and since no other problems arose, the questionnaire was not changed after the pilot study. 

### 2.4. Statistical Analysis

The normality of the distribution of the obtained data was verified using the Shapiro–Wilk test (which was chosen due to the size of the studied sample [41]), while the fish consumption in subgroups was compared using the Mann–Whitney U test and the correlations using the Spearman’s rank-order correlation. The accepted level of significance was *p* ≤ 0.05. 

The statistical analysis was conducted using Statgraphics Plus for Windows 5.1 (Statgraphics Technologies Inc., The Plains, VA, USA) and Statistica 8.0 (Statsoft Inc., Tulsa, OK, USA).

For subgroup analyses, all participants were divided into the following subgroups depending on:

- Gender: female and male (referring to the social context in which people live and which contributes to a subjective sexual identity, not to the anatomy of reproductive organs [42]);
- Age: younger (up to 16 years old) and older (>16 years old);
- Body mass index (BMI): underweight, proper, and excessive—for adults the standard cut-offs by WHO were applied (18.5–25 kg/m^2^ as proper body mass index) [43], while for minors the Polish growth reference cut-offs were applied [44] (5th–85th percentile as proper body mass index) [45];
- Locality of residence: countryside, small city (<50,000 inhabitants), medium city (50,000–100,000 inhabitants), and big city (>100,000 inhabitants);
- Region of residence: North-Western, Northern, Eastern, South-Eastern, Southern, Central, and Masovian—defined based on the macroregion categories assumed by the Central Statistical Office in Poland [46];
- Type of school: comprehensive school (comprehensive high schools and specialized high schools) and vocational school (vocational schools, technical schools, and visual arts high schools);
- The specific fish species declared to be usually consumed by the participants: salmon, cod, mackerel, herring, tuna, pollock, carp, rainbow trout (salmon trout), sprat, pangasius (basa), pike (luce), brook trout, zander (pikeperch), halibut, gilt-head bream, flounder, sole, perch, sardine, silver hake, eel, lemon sole; for this analysis only fish species that had been indicated by at least 10 participants were taken into consideration.

## 3. Results

The characteristics of the studied group are presented in Table 2. 

The frequency of declared fish and fish products intake of the studied group is presented in Table 3. Thirteen percent of participants declared to follow the recommendation to consume fish 1–2 times a week or more, while more than sixteen percent declared not to consume fish at all. Fish products were declared to be consumed less frequently than fish, and 4 in 10 participants declared not to consume fish products at all. 

Table 4 presents the weekly intake of total fish (fish and fish products) in the studied group, as well as in the various subgroups. The median intake was higher in males than in females (46.5 g vs. 31.6 g; *p* < 0.001) and higher in students from comprehensive schools compared to students from vocational schools (43.0 g vs. 29.0 g; *p* = 0.010). Only 6% (76 participants) met the recommendation to consume at least 300 g of fish weekly, while for the threshold of at least 150 g, it was 13% (170 participants) (data not presented in the table).

The total fish (fish and fish products) intake of the participants and their female guardians, and the correlation between them is presented in Table 5. The total fish intake of female guardians was declared to be higher among those whose child was female than of those whose child was male (58.1 g vs. 47.1 g; *p* = 0.009) (the *p*-value is not presented in the table). Also, the intake of female guardians of participants from comprehensive schools was declared to be higher than those of those from vocational schools in the whole group (75.6 g vs. 46.5 g; *p* < 0.001), as well as in the subgroup of female (79.1 g vs. 47.7 g; *p* < 0.001) and male (65.1 g vs. 40.7 g; *p* = 0.003) participants. The intake of the female guardian and the intake of the child were correlated in the whole group, as well as in all subgroups (*p* < 0.001).

Table 6 presents the weekly total fish (fish and fish products) intake of the participants and their male guardians and the correlation between them. Similarly to the case of the female guardians, the total fish intake of the male guardians was declared to be higher among those whose child was female than of those whose child was male (107.0 g vs. 93.0 g; *p* = 0.012) (the *p*-value is not presented in the table). Moreover, the intake of male guardians of participants from comprehensive schools was declared to be higher than of those from vocational schools in the whole group (125.6 g vs. 83.7 g; *p* < 0.001), as well as in the subgroup of female adolescents (119.1 g vs. 88.9 g; *p* = 0.005) and male adolescents (139.5 g vs. 65.1 g; *p* < 0.001). The intake of the male guardian and the intake of the child were correlated in the whole group, as well as in all subgroups (*p* < 0.001).

Table 7 presents the participants’ weekly total fish (fish and fish products) intake, the number of points from the fish knowledge test that the participants received, and the correlation between them. The median number of points from the fish knowledge test in the whole group was 10.0, and it did not differ among female and male adolescents (*p* = 0.136) (the *p*-value is not presented in the table). However, older participants received more points than the younger ones in the whole studied group (11.0 points vs. 9.0 points; *p* < 0.001) and in the subgroup of female adolescents (11.0 points vs. 9.0 points; *p* < 0.001), but not in the subgroup of male adolescents (*p* = 0.526). Moreover, students from comprehensive schools received more points than those from vocational schools in the whole studied group (11.0 points vs. 9.0 points; *p* < 0.001), in the subgroup of female individuals (11.0 points vs. 9.0 points; *p* < 0.001) and in the subgroup of male individuals (11.0 points vs. 9.0 points; *p* < 0.001). There was a correlation between the weekly total fish intake of the participants and the number of points they received from the fish knowledge test in the whole studied group (*p* < 0.001), but not in all of the subgroups. 

Table 8 presents the total fish (fish and fish products) intake in subgroups of participants stratified by the knowledge of the recommendation for children and adolescents to eat fish, as well as the recommended frequency of fish consumption. The intake was more than two times higher among students who knew that it is recommended that children and adolescents should eat fish (39.5 g vs. 18.6 g; *p* < 0.001) and also among those who knew that fish should be eaten more than once a week (58.1 g vs. 25.5 g; *p* < 0.001) compared to those who did not provide a correct answer to the analyzed statements.

The weekly total intake of fish (fish and fish products) in subgroups stratified by the specific fish species declared to be usually consumed by the participants is presented in Table 9. The total fish intake of participants who declared to usually consume cod, flounder, halibut, salmon, mackerel, pollock, silver hake, rainbow trout, sardine, pike, sprat, herring, and tuna was higher than that of those who declared to not usually consume these species. The biggest differences in fish intake were observed concerning consuming or not consuming salmon (3.5 times higher fish intake in the salmon-consuming group), cod, flounder, and rainbow trout (2.5 times higher fish intake in the consuming group), silver hake (2.4 times higher fish intake in the silver-hake-consuming group), and halibut and mackerel (2.3 times higher fish intake in the consuming group). 

## 4. Discussion

### 4.1. The Weekly Fish Intake 

According to the present study, the median weekly intake of fish and fish products among Polish youth corresponds to 34.9 g. This is much lower than the average apparent consumption in Poland based on food balance sheets being 256 g/week (13.33 kg yearly) [47], but it also seems to be lower than according to household budget surveys, which state that the average fish and seafood intake is 65 g/week (280 g monthly) [48]. This might indicate that the fish intake is lower among Polish youth than in the whole Polish population. However, in the present study, only questions about fish and fish products intake were posed, not about other seafood, while the food balance sheets and the household budget surveys concern all—fish and other seafood.

What should be underscored, however, is that in the present study, a possible underestimation bias is possible due to poor memory or the fact that some dishes containing fish might not be recognized as sources of fish in diet (e.g., fish casseroles, wraps with smoked salmon, or fish soup). On the other hand, the method used to estimate fish consumption in the study, a kind of simple version of the food frequency questionnaire (FFQ), usually tends to overestimate food intake [49].

Nevertheless, other research also shows that low fish intake is more frequent in children and adolescents than in adults [50,51]. In the USA, in the years 2013–2016, one in five adults consumed seafood (including fish) at least two times per week, while among youth aged 2 to 19 years, it was observed in only one in eighteen [50]. The 2005–2010 National Health and Nutrition Examination Surveys (NHANES) in the United Kingdom also indicated that the lowest fish consumption was observed among younger individuals [51]. Moreover, the German VeChi Youth Study indicated that the average fish intake in children and adolescents is often 0.0 g/day, even among omnivores [52].

Looking at the median fish consumption in the studied group, being 34.9 g/week, the fish intake among Polish youth needs to increase more than eight times to reach the recommended minimum fish intake of 300 g per week/capita [17,19]. However, more importantly, considering the proportion of individuals who do not reach the recommendations [17,19], 94% of Polish youth should increase their fish intake to at least 300 g weekly. Considering the two times lower threshold of the recommended weekly fish consumption, namely, 150 g [10,15], fish intake should be increased by 87% of participants to reach it. 

### 4.2. Differences in Fish Intake Depending on Gender

In the present study, male adolescents consumed almost 50% more fish and fish products than female adolescents (46.5 g vs. 31.6 g weekly). This is very much in line with the results from the German EsKiMo study according to which boys also consumed 50% more than girls—the median weekly intake among boys was 42 g, while among girls 28 g [53]. The higher fish intake among males, even though many studies indicate that women often have healthier food choices than men [54,55,56], might be due to the fact that men simply consume bigger food portions [57,58] and more energy [59], but also more animal protein [60] than women. 

Being an important source of essential nutrients such as vitamin D, iodine, selenium, and *n*-3 polyunsaturated fatty acids [6], fish and its consumption might play a role in reaching or not reaching their recommended intakes. Because the nutritional recommendations for the mentioned nutrients do not differ among genders [22], the results from the present study indicate that it might be easier for male youths to reach the recommendations owing to their higher fish consumption compared to females. It should also be noted, however, that even though in the present study the total fish intake was higher in male than in female participants, in both cases, it was very low. 

### 4.3. Frequency of Fish Intake

The recommendation to consume fish at least 1–2 times a week [10,15] in the present study was followed by only 13.0% of participants, while 16.5% of them declared not to consume fish at all. Nevertheless, the majority (70.2%) declared to consume fish only occasionally—three times a month or less. In a study conducted among 4–6-graders from a primary school in a coastal city in Poland, 44.5% of the pupils claimed to consume fish 1–2 times per week, 26.2% not to consume fish at all, and 14.1% less than once per week [61]. In a group of Polish 17–18-year-old females, only 8.7% reported frequent (2 to 3 times per week) fish consumption, 49.1% of them reported consuming fish once per month, and 42.2% once per week [62]. Moreover, in another study among Polish young adults aged 20–26, nine percent of them declared to consume fish and fish products several times a week, 31.5% once a week, 53.5% three times a month or less, and 6% declared not to consume fish at all. Interestingly, more men than women declared to consume them several times a week and once a week [55].

### 4.4. Fish Intake and Fish Knowledge

To increase fish intake, national educational campaigns concerning its benefits were introduced in Poland in the years 2008–2009 by the Ministry of Agriculture and Rural Development and were entitled ‘Fish affects everything’. According to the governmental data, an increase in fish and fish products intake was observed afterward—the annual mean fish intake in the year 2007 was 12.91 kg/person, while in the year 2008, it was 13.67 kg/person [63]. This is in line with the present study concerning the association between fish knowledge and fish intake. Adolescents who received more points from the fish knowledge test consumed more fish than their peers who received fewer points (*p* < 0.001). This was stated for the whole studied group as well as for almost all the analyzed subgroups. What might seem obvious is that older pupils’ knowledge was greater than the younger ones.

However, a less obvious observation was made concerning the type of school the participants attended. Those who attended comprehensive schools received more points than those from vocational schools. This is in line with the latest available data for the pass rate for the high school graduation exams in Poland, according to which 90% of pupils who attended comprehensive schools passed the exam, while in vocational schools in the same year, it was 79% [64]. This might indicate the fact that the level of education in Poland is on average higher in comprehensive schools compared to vocational schools, and the general education level probably influences the fish knowledge level. What should not be forgotten, however, is that the teaching in vocational schools focuses more on hands-on learning; hence, pupils from these schools are expected to have a wider knowledge concerning technical or professional issues.

What is more, when analyzing the two statements concerning fish consumption recommendations, it was stated that the median total fish intake was more than two times higher among those participants who knew that it is recommended that (1) children and adolescents should eat fish and that (2) fish should be eaten more than once a week. A recent intervention study from Indonesia indicated that nutritional education does increase fish consumption in children aged 10–12 [65], while a cross-sectional study from Italy and Croatia indicated that subjective knowledge concerning fish is an important predictor of fish consumption [66]. Similarly, according to the results from a consumer study conducted among adults living in Belgium, the Netherlands, Denmark, and Poland, subjective knowledge was a strong predictor of fish consumption [67]. Therefore, it can be supposed that nutritional education interventions aiming at improving adolescents’ knowledge of fish consumption and its benefits could also result in a higher fish intake in this vulnerable group. 

### 4.5. Determinants of Fish Intake

Children’s food choices depend highly on their parents’ food habits and thus food availability at home [68], whereas in the adolescence period, when independence and time spent with peers increases, social norms of food choice start to be more important [69] and influence the youth’s food intake [70]. The relationship between what children and adolescents eat with what their parents/legal guardians consume was observed in the present study, in which a correlation was seen for both male and female guardians. Hence, it seems that children’s fish intake is determined by their parents’/legal guardians’ fish intake. 

A crucial reason for this correlation might be fish accessibility at home. It is well known that food accessibility has a significant influence on its consumption when it comes to fruits and vegetables [71]; hence, it might also be one of the causes concerning fish. Another possible reason could be the extent of the early life exposure to fish and consequently developing a liking for fish or not developing it, which seems to be true for fruits [72]. Moreover, familiarity with fish might play a role in consuming or not consuming it. Having more opportunities to taste unfamiliar foods results in increased liking and consumption [73]; hence, parents who themselves consume fish and provide opportunities for their offspring to consume it too, might, owing to this, increase their children’s liking and consumption of fish. 

It is well known that food behaviors at home as well as early-life experiences concerning food taste and flavors have a significant influence on children’s future lives [74]. Other studies show a similar pattern concerning other food groups—providing fruit and vegetables at home and consuming fruit by the parents were associated with children’s healthy food choices such as consumption of fruit, vegetables, and whole grain cereal. On the other hand, parenting patterns such as allowing unhealthy snacks, providing an abundance of unhealthy foods at home, and rewarding with snacks and screen time was correlated with children’s higher consumption of snacks and fizzy drinks [68]. This highlights the importance of providing healthy, nutritious food products at home when the children are still small and can become accustomed to different tastes. Fish and fish products being consumed below the recommendations should be part of these food products.

Although it was hypothesized that other factors associated with fish intake in the youth would be their place of residence (region as well as the size of the locality), body mass index, and age, this was not the case in the studied group. Concerning the place of residence, Polish national household data on fish consumption indicate that while the average monthly consumption of fish and seafood in the whole country is 280 g/capita/month, for the different regions it varies from 210 g to 380 g [48]. This might indicate that there are regional differences in fish consumption. However, the data presented by Rogalińska et al. [48] do not specify whether the differences in fish consumption among the various regions are statistically significant or not. Moreover, it cannot be forgotten that household data do not take into consideration the fact that some of the purchased food is thrown away, or given to animals or guests, as well as that it is an average for all people living in a household, without differentiating that some of them might consume more than the others. In the UK National Diet and Nutrition Survey, which assessed oily fish intake across the lifespan, it was found that there was a substantial difference in fish intake between children, adolescents, and adults. While only 12.7% of children aged 4–11 and 13.2% of adolescents aged 12–19 were oily fish consumers, in the group of adults aged 30–39, the share was 26.5% [75]. This indicates that fish intake might increase with age, and therefore while the average fish and seafood consumption based on national household surveys is 280 g/capita/month [48], it is probably higher than that among older adults and lower among adolescents, who were surveyed in the present study.

When it comes to the size of the locality, some studies indicate that there is a correlation between the degree of urbanization and fish intake, as observed in the study by Klatka et al. [76], or that fish intake is higher in urban than in rural areas, as observed in the study by Petrenya [77]. However, although the study by Klatka et al. [76] concerned Polish adolescents and young adults aged 15–29 years old, only secondary data were used for the analyses, and the fish consumption data concerned the whole Polish population, which is not very precise. Moreover, an analysis of fish consumption drivers in different parts of the world found that while urbanization is an important fish consumption driver in poorer regions such as Africa and Bangladesh, it is not in others [78]. Therefore, the fact that in the present study, no differences in fish intake were observed concerning the size of the locality might result from the fact that Polish villages, and small, medium, and big cities do not differ from each other, which is the opposite to poorer parts of the world [78]. 

Surprisingly, in the present study, fish intake did not turn out to be associated with the participants’ body mass index. Even though one might believe that due to the numerous health benefits of eating fish [6,7,8,9], higher fish intake might be connected to having a proper body mass index, the results from the European Prospective Investigation into Cancer and Nutrition (EPIC) showed neither a relation between higher fish intake and lower body mass [79], nor between higher fish intake and waist circumference [80]. In the present study, another explanation of no differences might also simply be the fact that fish intake is in general very low among secondary school students. 

Apart from gender, which cannot be influenced, the two main determinants were their parents’/legal guardians’ fish intake and their knowledge of fish intake benefits and safety concerns. Since the fish intake of parents and legal guardians is challenging to alter, it seems to be crucial to focus on youth knowledge in the first place. 

In light of the results of the present study showing that fish intake among Polish youth is below the recommendations and that it is determined by parents’/legal guardians’ fish intake and the knowledge of fish consumption benefits and safety, and the indications that nutritional education can increase fish intake [63,65,81], more attention should be given to increasing the adolescents’ knowledge of fish consumption benefits. It should be addressed especially to young adolescents who consume less fish than male adolescents, as well as those who attend vocational schools. 

### 4.6. Fish Species Declared to Be Usually Consumed

Fish species that were the most commonly indicated, as well as discriminative for the total fish intake were salmon, cod, mackerel, herring, tuna, and pollock. Salmon, mackerel, and herring contain more than 10 g of fat in 100 g of fish [38], and thus are considered fatty (oily) fish, while cod, tuna, and pollock contain below 1.5 g of fat in 100 g [38], and are therefore considered lean (often called white) fish. Importantly, due to their higher *n*-3 fatty acids content, fatty fish are distinguished from the whole group of fish in numerous dietary recommendations [10,12,14,17].

In the present study, the most frequently chosen fish species was salmon (45% of participants declared to choose it), which, apart from containing significant amounts of *n*-3 fatty acids, also contains high amounts of vitamin D (13 μg/100 g) [38]. Moreover, what should be noted, salmon (wild and farmed) is one of the species containing very little mercury (≤0.1 μg/g) and very little-to-little dioxins (≤0.5 to 0.5–4 pg Toxic Equivalent (TEQ)/g depending on the type), and was classified to the first and first/second group, respectively, when it comes to the smallest content of those contaminants [82]. That is why the Food and Agriculture Organization (FAO) of the United Nations and the World Health Organization (WHO) recommended salmon as a fish species whose consumption has more benefits than risks [82]. Based on the above, it seems beneficial that, when consuming fish, many youths choose salmon, despite not reaching the recommended fish intake amounts. 

### 4.7. Limitations of the Study

Although the conducted study presents novel observations, some limitations of the study should be listed. Firstly, the acquired data were self-reported for adolescents and proxy-reported for their parents/legal guardians and retrospective, which may provide less accurate data than, e.g., a dietary record. However, due to the nationwide design of the study, as well as the fact that the collected data concerned fish, which is often not consumed on a daily basis, using a different method (e.g., 24 h dietary recall or the 3-day dietary record) would not have been possible (but it would be valuable to assess the fish intake while comparing it with the total daily intake of other food products). So as to minimize the inaccuracy of the data, much attention was given to the thorough preparation of questions and additional clues (e.g., highlighting which type of fish is asked in a specific question), as well as providing fish and fish products pictures with the corresponding weight to aid in the accurate estimation of fish and fish products intake. Moreover, the influence of confounding variables was not taken into account in the analyses, and the study must be seen as a descriptive one. 

Another limitation is that no questions were posed concerning the reasons for consuming or not consuming fish nor about being vegan or vegetarian, which might have enriched the results and helped establish recommendations for increasing fish consumption. However, making the questionnaire too long might have resulted in a lower response rate; hence, such questions were decided not to be asked. Last but not least, taking part in the study was voluntary; therefore, despite aiming for representativeness, due to an insufficient response rate within the study, the gathered data do not come from a representative sample. Nevertheless, it succeeded in gathering data from all macroregions of Poland, and the analysis was performed for more than 1300 adolescents.

Considering the importance as well as the novelty of the results from the present study, it would be worthwhile to conduct similar studies in countries with different dietary patterns. 

## 5. Conclusions

The median total fish consumption was 34.9 g/week, while 94% of participants did not meet the recommended fish intake of 300 g/week. Considering the two times lower threshold of 150 g/week, 87% of participants did not reach it. The fish consumption among males was higher than among females, and pupils from comprehensive schools reported higher intakes than those from vocational schools. The two main factors determining the participants’ total fish intake were their knowledge of fish intake benefits and safety concerns, and their parents’/legal guardians’ fish consumption. Nutritional education considering fish consumption benefits and recommendations is needed to increase fish intake in the vulnerable group of Polish youth.

## Figures and Tables

**Table 1:** Fish knowledge test concerning fish intake benefits and safety risks and the correct answers used in the study.

No.	Statement	Correct Answer
1.	Fish are a good source of protein.	True
2.	Fish contain a lot of fiber.	False
3.	Fish are a good source of vitamin D.	True
4.	Fish contain a lot of unhealthy fats.	False
5.	Fish have good fat.	True
6.	Eating fish is good for the heart.	True
7.	Eating fish is not good for the brain.	False
8.	Eating fish is good for you.	True
9.	Fish contain a lot of healthy ‘trans’ fats.	False
10.	Eating fish lowers cholesterol.	True
11.	Fish are a good source of vitamin C.	False
12.	Eating fish may cause allergies.	True
13.	Fish may contain bacteria or parasites.	True
14.	Children and adolescents should not eat fish.	False
15.	Eating fish is recommended to pregnant women.	True
16.	Fish may contain contaminants.	True
17.	Fish should be eaten once a week at the most.	False
18.	Fish may contain PCBs.	True
19.	Cod is a fatty fish.	False
20.	Fish may contain mercury.	True

**Table 2:** Characteristics of the studied group.

Characteristic		n (%)
Total		1317 (100)
Gender	Female	870 (66.1)
	Male	447 (33.9)
Region	North-Western	55 (4.2)
	Northern	329 (25.0)
	Eastern	99 (7.5)
	South-Western	378 (28.7)
	Southern	74 (5.6)
	Central	78 (5.9)
	Masovian	304 (23.1)
Place of residence	Countryside	681 (51.7)
	Small city (<50,000)	274 (20.8)
	Medium city (50,000–100,000)	136 (10.3)
	Big city (>100,000)	226 (17.2)
Body mass index	Underweight	96 (7.3)
	Proper body mass index	917 (69.6)
	Overweight or obese	304 (23.1)
Age	Older (>16 years old)	661 (50.2)
	Younger (up to 16 years old)	656 (49.8)
School	Comprehensive	462 (35.1)
	Vocational	855 (64.9)

**Table 3:** Frequency of declared fish and fish products intake of the studied group.

Categories of Frequency	Fish	Fish Products
	n (%)	n (%)
Never	217 (16.5)	537 (40.8)
<once a month	452 (34.3)	428 (32.5)
1–3 times a month	477 (36.2)	279 (21.2)
1–2 times a week	142 (10.8)	56 (4.3)
≥3 times a week	29 (2.2)	17 (1.3)

**Table 4:** The weekly intake of total fish (fish and fish products) in the studied group.

		Intake, Grams/Week		
Subgroups		Mean ± SD	Median (Min–Max)	*p*
Total		78.7 ± 135.8	34.9 (0.0–1650.0) *	–
Gender	Female	65.8 ± 108.9	31.6 (0.0–900.0) *	<0.001
	Male	103.6 ± 174.3	46.5 (0.0–1650.0) *	
Place of residence—region	North-Western	82.4 ± 123.8	40.7 (0.0–690.0) *	1.000
	Northern	76.3 ± 141.9	29.0 (0.0–1275.0) *	
	Eastern	64.2 ± 125.4	17.4 (0.0–808.0) *	
	South-Western	85.1 ± 138.2	34.9 (0.0–900.0) *	
	Southern	63.1 ± 83.8	34.0 (0.0–405.0) *	
	Central	80.1 ± 91.4	56.4 (0.0–495.0) *	
	Masovian	80.7 ± 150.7	39.5 (0.0–1650.0) *	
Place of residence—size	Countryside	68.5 ± 105.9	32.0 (0.0–750.0) *	
	Small city (<50,000)	90.5 ± 157.6	39.5 (0.0–1650.0) *	0.567
	Medium city (50,000–100,000)	71.3 ± 112.7	40.7 (0.0–900.0) *	
	Big city (>100,000)	99.4 ± 187.8	37.1 (0.0–1275.0) *	
Body mass index	Underweight	77.6 ± 127.9	30.2 (0.0–675.0) *	0.407
	Proper body mass index	75.0 ± 130.9	38.3 (0.0–1650.0) *	
	Overweight or obese	90.2 ± 151.7	31.9 (0.0–900.0) *	
Age	Older (>16 years old)	82.4 ± 142.6	34.9 (0.0–1650.0) *	0.666
	Younger (up to 16 years old)	74.9 ± 128.6	34.9 (0.0–1275.0) *	
School	Comprehensive	86.4 ± 134.6	43.0 (0.0–1200.0) *	0.010
	Vocational	74.5 ± 136.3	29.0 (0.0–1650.0) *	

* non-parametric distribution (verified using Shapiro–Wilk test; *p* ≤ 0.05).

**Table 5:** The weekly total fish (fish and fish products) intake of the participants and their female guardians and the correlation between them.

Subgroups			Intake—Child, Grams/Week		Intake—Female Guardian, Grams/Week		Female Guardians’ Intake Differences in Subgroups	Correlation Child–Female Guardian	
			Mean ± SD	Median (Min–Max)	Mean ± SD	Median (Min–Max)	*p*	R	*p*
All (female and male adolescents) (n = 1256)	Total		77.6 ± 131.8	34.9 (0.0–1650.0) *	107.2 ± 158.5	55.8 (0.0–1575.0) *	-	0.4563	<0.001
	Age	Younger (up to 16 years old) (n = 630)	73.5 ± 122.0	34.9 (0.0–1275.0) *	101.6 ± 151.6	47.7 (0.0–1575.0) *	0.256	0.4442	<0.001
		Older (>16) (n = 626)	81.6 ± 140.9	34.9 (0.0–1650.0) *	113.0 ± 165.1	58.7 (0.0–1332.0) *		0.4675	<0.001
	School	Comprehensive (n = 451)	83.6 ± 121.4	43.0 (0.0–900.0) *	119.7 ± 151.4	75.6 (0.0–1575.0) *	<0.001	0.4795	<0.001
		Vocational (n = 805)	74.2 ± 137.2	26.7 (0.0–1650.0) *	100.3 ± 162.1	46.5 (0.0–1332.0) *		0.4361	<0.001
Female adolescents (n = 844)	Total		66.0 ± 107.6	32.0 (0.0–900.0) *	110.8 ± 149.3	58.1 (0.0–1275.0) *	-	0.4886	<0.001
	Age	Younger (up to 16 years old) (n = 387)	60.6 ± 102.3	29.1 (0.0–900.0) *	104.9 ± 132.5	58.1 (0.0–750.0) *	0.995	0.4914	<0.001
		Older (>16 years old) (n = 457)	70.6 ± 111.8	33.1 (0.0–900.0) *	115.9 ± 162.1	60.5 (0.0–1275.0) *		0.4852	<0.001
	School	Comprehensive (n = 338)	75.1 ± 119.0	39.5 (0.0–900.0) *	122.4 ± 143.5	79.1 (0.0–787.5) *	0.001	0.5058	<0.001
		Vocational (n = 506)	60.0 ± 98.9	23.3 (0.0–900.0) *	103.1 ± 152.7	47.7 (0.0–1275.0) *		0.4642	<0.001
Male adolescents (n = 412)	Total		101.2 ± 168.7	46.5 (0.0–1650.0) *	99.8 ± 175.9	47.1 (0.0–1575.0) *	-	0.4358	<0.001
	Age	Younger (up to 16 years old) (n = 243)	94.0 ± 146.1	45.3 (0.0–1275.0) *	96.3 ± 178.0	45.3 (0.0–1575.0) *	0.164	0.4200	<0.001
		Older (>16) (n = 169)	111.6 ± 196.7	49.3 (0.0–1650.0) *	105.0 ± 173.2	58.1 (0.0–1332.0) *		0.4479	<0.001
	School	Comprehensive (n = 113)	109.2 ± 125.4	74.4 (0.0–600.0) *	111.5 ± 173.1	65.1 (0.0–1575.0) *	0.003	0.4365	<0.001
		Vocational (n = 299)	98.2 ± 182.5	39.5 (0.0–1650.0) *	95.4 ± 177.0	40.7 (0.0–1332.0) *		0.4280	<0.001

* non-parametric distribution (verified using Shapiro–Wilk test; *p* ≤ 0.05).

**Table 6:** The weekly total fish (fish and fish products) intake of the participants and their male guardians and the correlation between them.

Subgroups			Intake—Child, Grams/Week		Intake—Male Guardian, Grams/Week		Male Guardians’ Intake Differences in Subgroups	Correlation Child–Male Guardian	
			Mean ± SD	Median (Min–Max)	Mean ± SD	Median (Min–Max)	*p*	R	*p*
All (female and male adolescents) (n = 1029)	Total		76.4 ± 118.3	38.3 (0.0–1200.0) *	165.7 ± 213.7	102.3 (0.0–1800.0) *	–	0.4084	<0.001
	Age	Younger (up to 16 years old) (n = 525)	74.1 ± 115.4	39.5 (0.0–1200.0) *	157.1 ± 192.8	101.7 (0.0–1140.0) *	0.423	0.4072	<0.001
		Older (>16) (n = 504)	78.9 ± 121.4	37.1 (0.0–833.2) *	174.7 ± 233.3	107.0 (0.0–1800.0) *		0.4070	<0.001
	School	Comprehensive (n = 380)	89.0 ± 135.7	44.7 (0.0–1200.0) *	180.8 ± 194.0	125.6 (0.0–1275.0) *	<0.001	0.4274	<0.001
		Vocational (n = 649)	69.1 ± 106.3	31.3 (0.0–840.0) *	156.9 ± 224.1	83.7 (0.0–1800.0) *		0.3913	<0.001
Female adolescents (n = 670)	Total		65.0 ± 100.8	32.0 (0.0–900.0) *	176.3 ± 223.5	107.0 (0.0–1410.0) *	-	0.3949	<0.001
	Age	Younger (up to 16 years old) (n = 316)	58.6 ± 89.3	31.9 (0.0–900.0) *	164.9 ± 203.7	102.3 (0.0–1140.0) *	0.407	0.4136	<0.001
		Older (>16 years old) (n = 354)	70.8 ± 109.9	32.8 (0.0–750.0) *	186.5 ± 239.7	108.1 (0.0–1410.0) *		0.3745	<0.001
	School	Comprehensive (n = 282)	77.9 ± 121.8	41.9 (0.0–900.0) *	181.0 ± 199.1	119.1 (0.0–1275.0) *	0.005	0.4249	<0.001
		Vocational (n = 388)	55.7 ± 81.2	23.3 (0.0–555.0) *	173.0 ± 239.9	88.9 (0.0–1410.0) *		0.3650	<0.001
Male adolescents (n = 359)	Total		97.7 ± 143.2	52.3 (0.0–1200.0) *	145.9 ± 192.6	93.0 (0.0–1800.0) *	-	0.4626	<0.001
	Age	Younger (up to 16 years old) (n = 209)	97.5 ± 143.4	51.1 (0.0–1200.0) *	145.2 ± 174.7	101.1 (0.0–1125.0) *	0.844	0.4316	<0.001
		Older (>16) (n = 150)	98.0 ± 143.4	57.5 (0.0–833.2) *	146.8 ± 215.6	87.5 (0.0–1800.0) *		0.5118	<0.001
	School	Comprehensive (n = 98)	120.9 ± 166.2	79.1 (0.0–1200.0) *	180.4 ± 179.3	139.5 (0.0–1125.0) *	<0.001	0.4265	<0.001
		Vocational (n = 261)	89.0 ± 132.9	43.5 (0.0–840.0) *	132.9 ± 196.1	65.1 (0.0–1800.0) *		0.4648	<0.001

* non-parametric distribution (verified using Shapiro–Wilk test; *p* ≤ 0.05).

**Table 7:** The participants’ weekly total fish (fish and fish products) intake, the number of points from the fish knowledge test, and the correlation between them.

Subgroups			Fish Intake, Grams/Week		Fish Knowledge Test (Points, Max = 20)		Fish Knowledge Test Differences in Subgroups	Correlation Fish Intake–Fish Knowledge	
			Mean ± SD	Median (Min–Max)	Mean ± SD	Median (Min–Max)	*p*	R	*p*
All (female and male adolescents)	Total (n = 1317)		78.7 ± 135.8	34.9 (0.0–1650.0) *	9.5 ± 4.4	10.0 (0.0–20.0) *	–	0.1884	<0.001
	Age	Younger (up to 16 years old) (n = 525)	74.9 ± 128.6	34.9 (0.0–1275.0) *	9.0 ± 4.4	9.0 (0.0–19.0) *	<0.001	0.4072	0.201
		Older (>16) (n = 504)	82.4 ± 142.6	34.9 (0.0–1650.0) *	9.9 ± 4.3	11.0 (0.0–20.0) *		0.4070	0.176
	School	Comprehensive (n = 380)	86.4 ± 134.6	43.0 (0.0–1200.0) *	10.6 ± 4.1	11.0 (0.0–19.0) *	<0.001	0.4274	0.181
		Vocational (n = 649)	74.5 ± 136.3	29.0 (0.0–1650.0) *	8.8 ± 4.4	9.0 (0.0–20.0) *		0.3913	0.180
Female adolescents	Total (n = 870)		65.8 ± 108.9	31.6 (0.0–900.0) *	9.7 ± 4.2	10.0 (0.0–20.0) *	-	0.1913	<0.001
	Age	Younger (up to 16 years old) (n = 316)	59.4 ± 101.2	28.4 (0.0–900.0) *	9.0 ± 4.2	9.0 (0.0–19.0) *	<0.001	0.2178	<0.001
		Older (>16 years old) (n = 354)	71.2 ± 114.8	32.8 (0.0–900.0) *	10.2 ± 4.1	11.0 (0.0–20.0) *		0.1678	<0.001
	School	Comprehensive (n = 282)	76.2 ± 123.7	39.5 (0.0–900.0) *	10.6 ± 4.1	11.0 (0.0–19.0) *	<0.001	0.2064	<0.001
		Vocational (n = 388)	59.0 ± 97.5	23.3 (0.0–900.0) *	9.0 ± 4.1	9.0 (0.0–20.0) *		0.1584	<0.001
Male adolescents	Total (n = 447)		103.7 ± 174.3	46.5 (0.0–1650.0) *	9.1 ± 4.7	10.0 (0.0–20.0) *	-	0.2009	<0.001
	Age	Younger (up to 16 years old) (n = 209)	98.8 ± 159.4	45.9 (0.0–1275.0) *	8.9 ± 4.7	10.0 (0.0–18.0) *	0.526	0.1803	0.004
		Older (>16) (n = 150)	110.3 ± 193.0	47.7 (0.0–1650.0) *	9.2 ± 4.8	10.0 (0.0–20.0) *		0.2267	0.002
	School	Comprehensive (n = 98)	116.0 ± 159.3	74.4 (0.0–1200.0) *	10.6 ± 4.0	11.0 (0.0–18.0) *	<0.001	0.1222	0.188
		Vocational (n = 261)	99.2 ± 179.4	40.6 (0.0–1650.0) *	8.5 ± 4.8	9.0 (0.0–20.0) *		0.2214	<0.001

* non-parametric distribution (verified using Shapiro–Wilk test; *p* ≤ 0.05).

**Table 8:** Total weekly total fish (fish and fish products) intake in subgroups stratified by the knowledge of the recommendation for children and adolescents to eat fish, as well as the recommended frequency of fish.

Analyzed Knowledge of Fish Consumption Recommendations	Correctness of Answers	Intake, Grams/Week		*p*
		Mean ± SD	Median (Min–Max)	
‘Children and adolescents should eat fish’	Correct answer (n = 991)	82.8 ± 129.5	39.5 (0.0–127.0) *	<0.001
	No correct answer (n = 326)	66.2 ± 152.9	18.6 (0.0–1650.0) *	
‘Fish should be eaten more than once a week’	Correct answer (n = 439)	113.2 ± 161.1	58.1 (0.0–1275.0) *	<0.001
	No correct answer (n = 878)	61.4 ± 117.6	25.5 (0.0–1650.0) *	

* non-parametric distribution (verified using Shapiro–Wilk test; *p* ≤ 0.05).

**Table 9:** The weekly total fish (fish and fish products) intake in subgroups stratified by the specific fish species usually consumed by the participants.

Fish Species (n^c^/n^n^)	Total Fish Intake, Grams/Week				*p*
	Consuming the Given Fish Species		Not Consuming the Given Fish Species		
	Mean ± SD	Median (Min–Max)	Mean ± SD	Median (Min–Max)	
Salmon (590/727)	103.2 ± 137.5	56.9 (0.0–1275.0) *	58.7 ± 131.2	16.2 (0.0–1650.0) *	<0.001
Cod (410/907)	93.8 ± 114.5	58.1 (0.0–750.0) *	71.8 ± 143.9	23.2 (0.0–1650.0) *	<0.001
Mackerel (305/1012)	101.7 ± 129.9	58.1 (0.0–750.0) *	71.7 ± 136.9	25.5 (0.0–1650.0) *	<0.001
Herring (238/1079)	103.8 ± 135.7	58.1 (0.0–900.0) *	73.1 ± 135.3	29.0 (0.0–1650.0) *	<0.001
Tuna (207/1110)	93.8 ± 119.7	55.8 (0.0–750.0) *	75.8 ± 138.5	32.0 (0.0–1650.0) *	<0.001
Pollock (189/1128)	100.4 ± 144.8	51.1 (0.0–1200.0) *	75.0 ± 134.0	31.9 (0.0–1650.0) *	<0.001
Carp (137/1180)	78.7 ± 124.3	34.9 (0.0–900.0) *	78.7 ± 137.1	34.9 (0.0–1650.0) *	0.137
Rainbow trout (salmon trout) (75/1242)	141.0 ± 164.4	83.7 (0.0–840.0) *	74.9 ± 133.0	33.1 (0.0–1650.0) *	<0.001
Sprat (69/1248)	100.0 ± 165.7	46.5 (0.6–900.0) *	77.5 ± 133.9	34.8 (0.0–1650.0) *	0.006
Pangasius (basa) (66/1251)	52.5 ± 56.2	29.1 (0.0–270.0) *	80.0 ± 138.6	34.9 (0.0–1650.0) *	0.889
Pike (luce) (56/1261)	94.2 ± 126.0	55.2 (0.0–630.0) *	78.0 ± 136.2	34.9 (0.0–1650.0) *	0.035
Brook trout (54/1263)	99.6 ± 129.0	45.3 (0.1–525.0) *	77.8 ± 136.1	34.9 (0.0–1650.0) *	0.062
Zander (pikeperch) (49/1268)	59.9 ± 59.0	41.9 (0.0–339.8) *	79.4 ± 137.9	34.9 (0.0–1650.0) *	0.194
Halibut (46/1271)	92.4 ± 91.5	80.2 (0.0–450.0) *	78.2 ± 137.1	34.9 (0.0–1650.0) *	0.003
Gilt-head bream (32/1285)	117.1 ± 194.8	47.1 (0.0–808.0) *	77.7 ± 134.0	34.9 (0.0–1650.0) *	0.132
Flounder (29/1288)	98.8 ± 108.5	87.2 (0.5–450.0) *	78.2 ± 136.4	34.9 (0.0–1650.0) *	0.016
Sole (29/1288)	77.7 ± 83.2	43.0 (0.0–300.0) *	78.7 ± 136.8	34.9 (0.0–1650.0) *	0.137
Perch (28/1289)	131.8 ± 269.4	38.4 (0.0–1200.0) *	77.5 ± 131.4	34.9 (0.0–1650.0) *	0.272
Sardine (27/1290)	145.2 ± 217.5	44.1 (1.4–900.0) *	77.3 ± 133.3	34.9 (0.0–1650.0) *	0.049
Silver hake (23/1294)	147.3 ± 210.7	83.7 (0.0–808.0) *	77.4 ± 133.9	34.9 (0.0–1650.0) *	0.027
Eel (15/1302)	79.9 ± 96.9	47.7 (0.0–300.0) *	78.7 ± 136.2	34.9 (0.0–1650.0) *	0.615
Lemon sole (3/1314)	26.4 ± 45.6	0.0 (0.0–79.1) *	78.8 ± 135.9	34.9 (0.0–1650.0) *	0.200

n^c^—the number of participants who declared to consume the given fish species; n^n^—the number of participants who declared not to consume the given fish species; * non-parametric distribution (verified using Shapiro–Wilk test; *p* ≤ 0.05).

## Data Availability

The data presented in this study are available on request from the corresponding author. The data are not publicly available due to privacy.
